# Tumor-derived sphingosine-1-phosphate shapes angiogenesis in the acidic microenvironment of osteosarcoma via paracrine and autocrine signaling

**DOI:** 10.3389/fcell.2026.1831997

**Published:** 2026-05-21

**Authors:** Sofia Avnet, Nicolò Bozzini, Phuong-Nhi Nguyen, Maria Veronica Lipreri, Francesca Perut, Nicola Baldini, Margherita Cortini

**Affiliations:** 1 Department of Biomedical and Neuromotor Sciences, Alma Mater Studiorum, Università di Bologna, Bologna, Italy; 2 Biomedical Science, Technology and Nanobiotechnology Laboratory, IRCCS Istituto Ortopedico Rizzoli, Bologna, Italy

**Keywords:** 3D tumor models, acidosis, angiogenesis, osteosarcoma, sphingosine-1-phosphate, tumor microenvironment

## Abstract

**Introduction:**

Tumor-associated angiogenesis is a critical driver of tumor progression and is frequently characterized by excessive branching and structural disorganization. These abnormalities arise from dynamic interactions between tumor cells and the microenvironment, where metabolic stressors such as hypoxia and extracellular acidosis promote the release of pro-angiogenic factors. Among these, sphingosine-1-phosphate (S1P) has emerged as a key bioactive lipid involved in vascular development. We previously demonstrated that acidosis promotes sphingomyelin turnover and S1P secretion in osteosarcoma cells, enhancing tumor cell survival and migration. In this study, we investigated the role of S1P in osteosarcoma-associated angiogenesis and the contribution of tumor acidosis to this process.

**Methods:**

Matrigel®-based angiogenesis assays, HUVEC cultures, 3D osteosarcoma spheroids, and microfluidic systems were employed to evaluate endothelial sprouting and tubulogenesis. S1P signaling was pharmacologically inhibited using the FDA-approved S1P modulator FTY720 (Fingolimod). Conditioned media from osteosarcoma spheroids cultured under neutral or acidic conditions were analyzed for their pro-angiogenic activity. Soluble and extracellular vesicle-associated angiogenic mediators were also assessed.

**Results:**

S1P dose-dependently impaired endothelial tubulogenesis while strongly promoting endothelial sprouting. Conditioned media derived from acid-stimulated osteosarcoma spheroids significantly increased endothelial tubule length and branching compared with conditioned media from spheroids maintained at neutral pH. These effects were markedly reduced by FTY720 treatment. Furthermore, tumor-derived S1P activated autocrine signaling in osteosarcoma cells, enhancing the secretion of soluble and extracellular vesicle-associated pro-angiogenic mediators, including bFGF and the TGF-β co-receptor Endoglin (CD105).

**Discussion:**

These findings identify a previously unrecognized acidosis–S1P axis that contributes to angiogenic remodeling in osteosarcoma. Our results highlight the multifaceted role of S1P in regulating endothelial behavior and suggest that targeting S1P signaling may represent a promising strategy to disrupt pathological neoangiogenesis in osteosarcoma.

## 1 Introduction

Angiogenesis is a fundamental process in tumor progression and metastasis. Rather than uniformly expanding through vessel elongation, tumor vasculature is typically characterized by excessive branching, tortuosity, and profound structural disorganization. This aberrant architecture gives rise to irregular vessel paths and a chaotic network that sharply contrasts with normal vasculature (Majidpoor and Mortezaee, 2021; Ramjiawan et al., 2017). Tumor vascularization is strongly influenced by the tumor microenvironment (TME), which promotes the release of pro-angiogenic factors by tumor cells (Kaur and Roy, 2024; Liu et al., 2023). The TME is a dynamic and heterogeneous milieu consisting of non-malignant cell types, including immune cells, fibroblasts, endothelial cells, adipocytes, and mesenchymal stromal cells (MSC), alongside extracellular matrix (ECM) components and a complex network of signaling molecules. In addition, different availability and gradient of metabolites in the TME contribute to shape tumor cells adaptation and aggressiveness through a metabolic reprogramming characterized by enhanced glycolysis, oxygen deprivation, and accumulation of acidic metabolic byproducts, collectively contributing to a persistently acidic extracellular environment acidosis (Cortini et al., 2017; de Visser and Joyce, 2023; Jin and Jin, 2020; Li et al., 2025). Altogether, this metabolic stress activates transcriptional pathways, including hypoxia-inducible factor-1α (HIF-1α) and stimulate the release of key angiogenic mediators by the cells of the TME, such as vascular endothelial growth factor (VEGF), fibroblast growth factor (FGF), platelet-derived growth factor (PDGF), and angiopoietins, which promote endothelial cell proliferation, migration, and vascular remodeling (Jiang et al., 2020; Lugano et al., 2020; Nyberg et al., 2008).

Among the central mediators of angiogenesis is sphingosine-1-phosphate (S1P), a bioactive lipid that plays a crucial role in both physiological and pathological neovascularization. S1P regulates endothelial cell migration, tubule formation, and vessel stabilization (Avnet et al., 2025; Chae et al., 2004; Lee et al., 1999; Wang et al., 2019), primarily through signaling via five specific G protein-coupled receptors (S1PR_1–5_) (Avnet et al., 2025; Wang et al., 2019; Hisano and Hla, 2019). However, the biological effects of S1P are highly context-dependent, modulated by local concentration gradients and receptor subtypes (Wang et al., 2019; Hisano and Hla, 2019). In particular, pro-angiogenic effects are largely mediated by the S1PR_1_ and S1PR_3_ receptors, whereas S1PR_2_ typically conveys anti-angiogenic signals (Avnet et al., 2025; Takuwa et al., 2010).

In osteosarcoma (OS), the most frequent primary malignant bone tumor, predominantly affecting children and adolescents (Beird et al., 2022), we and others have found that elevated S1P promotes tumor cell survival and migration through multiple mechanisms (Pyne et al., 2018; Pyne and Pyne, 2020), including signaling via S1PR_3_-and S1PR_4_/STAT3 (Signal Transducer and Activator of Transcription 3) (Li et al., 2024; Shen et al., 2019), or by activating Baculoviral IAP Repeat-Containing protein (BIRC) and Tumor Necrosis Factor (TNF) Receptor-Associated Factor (TRAF), when tumor cells are cultured under acidosis, leading to stimulation of the non-canonical Nuclear Factor kappa-light-chain-enhancer of activated B cells (NF-kB) pro-survival pathway (Avnet et al., 2019; Cortini et al., 2021). We have also previously demonstrated that extracellular acidosis in the OS TME induces metabolic adaptations characterized by increased sphingomyelin turnover and elevated S1P synthesis and secretion (Cortini et al., 2021).

Notably, mounting evidence supports a direct role for acidosis in the regulation of angiogenesis (Perut et al., 2019; Rastogi et al., 2023; Thews and Riemann, 2019), and given our previous findings, it is possible to speculate the existence of a feed-forward loop in which tumor acidosis and angiogenesis create a pro-tumorigenic vicious circle through S1P-mediated signaling. The study of the existence of this complex regulatory network, dependent on S1P concentration, receptor expression, and extracellular pH, may thus further reveal the therapeutic potential of targeting the S1P axis. Clinically, this is particularly relevant in OS, where diverse progression and poor prognosis have been closely associated with increased microvascular density, underscoring the central role of angiogenesis in tumor development (Liu et al., 2023; Zhang et al., 2024). Targeting this axis may represent a promising anti-angiogenic strategy adapted to the unique metabolic landscape of OS (Hisano and Hla, 2019; Li et al., 2024; Shen et al., 2019).

Based on these premises, in this study we investigated the specific contribution of S1P in OS-associated angiogenesis, with a focus on its stage-specific effects during vascular morphogenesis. Using *in vitro* angiogenesis assays, 3D tumor spheroid models, and pharmacological inhibitors, we demonstrated that S1P selectively promotes endothelial sprouting without enhancing tubulogenesis. Furthermore, we showed that tumor-derived S1P, enhanced by extracellular acidosis, acts through autocrine signaling to further stimulate angiogenesis by promoting the release of additional pro-angiogenic mediators, both in soluble form and within extracellular vesicles.

## 2 Materials and methods

### 2.1 Cell lines

Human umbilical vein endothelial cells (HUVEC-GFP) were obtained from PromoCell (Heidelberg, Germany) and cultured in Endothelial Cell Basal Medium (ECBM) (PromoCell) supplemented with Endothelial Cell Growth Medium Supplement Pack (PromoCell), 8% Fetal Bovine Serum (FBS) (Lonza, Basil, Switzerland) and 1% Penicillin/Streptomycin (EuroClone, Milan, Italy). HUVEC were cultured on gelatin-coated flasks (0.2% in PBS). 143B cell were purchased from the American Type Culture Collection (ATCC, Washington, DC, United States) and cultured in IMDM (Life Technologies, Carlsbad, CA, United States) supplemented with 10% FBS (Lonza) and 1% Penicillin/Streptomycin (EuroClone). AD-MSC-GFP cells were kindly provided by Dr. Massimo Dominici (Modena, Italy); the transfection protocol was previously described (Avnet et al., 2021). All cells were cultured at 37 °C in a humidified atmosphere with 5% CO_2_, tested against mycoplasm and 143B cells validated (LGC Standards, Milan, Italy) in 2024. ADMSC-GFP and HUVEC cells were used between passages 3 and 9 and were used at 70%–80% confluence for experimental seeding.

To generate OS-ADMSC-GFP spheroids, OS cells were co-cultured with AD-MSC, as previuosly described (Cortini et al., 2023) and grown under two different conditions in 96-well ultra-low attachment round-bottom plates (S-BIO, NH, United States). For acid-grown spheroids, RPMI medium was used without NaHCO_3_ supplementation and therefore without bicarbonate buffering. Under these conditions, medium pH was allowed to vary dynamically in response to incubator CO_2_ levels and spheroid-dependent factors, including cell density, growth, and metabolic activity. Medium pH was monitored over time, and, accordingly, progressively decreased from approximately 6.8 to 6.5 over 96 h, in agreement with our previuos results (Bozzini et al., 2026), and partially recapitulating the acidic TME *in vivo* (Avnet et al., 2021; Gillies et al., 1994). These conditions likely shaped the intraspheroid microenvironment, particularly the intercellular compartment, through the combined effects of CO_2_ (Hulikova and Swietach, 2014), excess extracellular protons, and local metabolic activity, partially recapitulating the acidic TME *in vivo*. 143B and ADMSC-GFP cells were seeded at a 1:3 ratio, specifically 5 × 10^3^ 143B cells and 1.5 × 10^4^ cells/well in 200 μL of medium/well and resulting spheroids were then cultured for 5 days. 24 h after seeding, spheroids were treated with FTY720 0.3 μM (Merck, Darmstadt, Germany) or with S1P at the following concentrations: 2.5–25–100–250–500–1000–3000 nM (Merck).

### 2.2 Proliferation assay

HUVECs were seeded in 96-well flat-bottom tissue-culture plates pre-coated with 0.2% gelatin. Proliferation was monitored up to 72 h in the presence or absence of 250 nM S1P. To quantify total cell number, cultures were stained with Hoechst 33342 (2.25 μg/mL) for 20 min in complete medium. Fluorescent images were acquired using an ImageXpress Pico Automated Cell Imaging System (Molecular Devices, San Jose, CA, United States) with a 4× objective. Cell counts were obtained by automated detection of Hoechst-positive nuclei. Three independent replicates were performed.

### 2.3 Tubulogenesis assay

For tubulogenesis assays, 96-well, flat-bottom tissue culture plates coated with 60 μL/well of Matrigel® matrix growth factor reduced (Corning, New York, United States) mixed at 70% Matrigel +30% supplemented ECBM. HUVEC-GFP cells were counted and seeded on top at a density of 1.5 × 10^4^ cells/well. For conditioned medium (CM) experiments, spheroid supernatants were collected after 5 days of cell culture and used at 50% for tubulogenesis assays (50% CM + 50% endothelial cell medium). Alternatively, HUVEC-GFP cells were incubated with FTY720 0.3 μM or S1P at the following concentrations: 2.5-25-100-250-500-1000-3000 nM. Tubulogenesis was imaged by time-lapse microscopy for 24 h and tubules were quantified between 12 and 15 h post-seeding. Acquisition was obtained with the ImageXpress Pico; green (GFP) and brightfield emissions were measured. Capillary tube-like length and number of nodes were quantified using ImageJ software. Four independent replicates were performed.

### 2.4 Sprouting assay

Sprouting assay was performed as previously described (Avnet et al., 2025). A single-cell suspension of HUVEC-GFP cells (2 × 10^4^ cells/channel) was prepared from a 70% confluent flask following trypsinization and counting. Before seeding, the middle lane of a three-channel Mimetas® device (Mimetas, Oegstgeest, Netherlands) was filled with a matrix mixture composed of 60% Matrigel® (Corning), 30% neutralized Rat Tail Collagen I (30 mg/mL, Merck), and 10% endothelial medium (PromoCell). HUVEC-GFP cells were then introduced into the upper channel and allowed to form vessels over 24 h, as previously described (Avnet et al., 2025). Vessel formation was evaluated by live GFP confocal imaging. Subsequently, an angiogenic stimulus (37.5 ng/mL VEGF in the presence or absence of 250 nM S1P, Merck) was applied to the lower channel. The device was placed on a rocker set to alternate its tilt every 8 min to mimic perfusion flow. After further 24 h, vessels were imaged again by confocal microscopy (Nikon A1R MP; 20× air objective; 3 μm z-steps across a 180 μm z-stack) (Ni-E ZDrive, Nikon, Minato, Japan) and maximum-intensity projections or 3D reconstructions of the middle channel were generated. Images were quantified with NIS Elements AR 5.40.01. Sprouting was measured and quantified using maximum intensity projection images, excluding cells on top of the phase guide. Three independent replicates were performed.

### 2.5 ELISA

To obtain supernatants, OS-ADMSC-GFP spheroids were cultured under neutral or unbuffered acidic conditions and treated with FTY720. ELISA protocols for bFGF (R&D Systems, Minneapolis, MA, United States), VEGF (R&D Systems) and EGF (Bio-Techne, Minneapolis, Minnesota, United States) were followed according to manufacturer’s instruction.

For S1P ELISA, cell supernatants were concentrated using Vivaspin® 6 Centrifugal Concentrator (Sartorius, Göttingen, Germany) to increase S1P concentration. ELISA were performed immediately after collection, to minimize S1P degradation due to freeze/thaw cycles. S1P was measured in cell supernatants with the Sphingosine 1-Phosphate Assay Kit (Echelon, San Jose, CA, United States) according to the manufacturer’s instructions.

All data were normalized to total protein content in the supernatants, measured by BCA Protein Assay (Pierce, Waltham, MA, United States). All ELISAs were performed from two independent biological replicates.

### 2.6 RNA extraction, and gene expression

RNA was extracted using TRIzol® reagent (ThermoFisher, Waltham, MA, United States). Total RNA was reverse-transcribed into cDNA with MuLV Reverse Transcriptase, RNase inhibitor (Applied Biosystems, Foster City, CA, United States) and using random hexamers for first-strand synthesis. qPCR was performed on 500 ng of cDNA with the SsoAdvanced SYBR Green Mix on a CFX96 Touch system (Bio-Rad, Hercules, CA, United States). Primer sequences were as follows: GAPDH For: cca​agg​agt​aag​acc​cct​gg; GAPDH Rev: agg​gga​gat​tca​gtg​tgg​tg; Gusb For: ccc​act​cag​tag​cca​agt​ca; Gusb Rev: gtt​ctg​ctg​ctg​tgg​aag​tc; YWHZ For: ccg​cat​gat​ctt​tct​ggc​tc; YWHZ Rev: tag​tct​gtg​gga​tgc​aag​ca; S1PR1 For: cca​aga​aat​tcc​acc​gac​cc; S1PR1 Rev: ccc​cag​aca​aga​gca​ggt​ta; S1PR2 For: gga​gta​cct​gaa​ccc​caa​ca; S1PR2 Rev: cgc​aac​aga​gga​tga​cga​tg; S1PR3 For: gtg​ctc​ggc​cag​tta​caa​aa; S1PR3 Rev: tga​cag​cga​ggg​ttt​gtt​tg; S1PR4 For: cgc​ttc​tgt​gtg​att​ctg​gg; S1PR4 Rev: tcg​aac​ttc​aat​gtt​gcc​agg; S1PR5 For: gag​gac​tca​ggc​taa​ggt​gg; S1PR5 Rev: tga​ttc​gga​ggg​gtc​ttc​ag. Gene expression was normalized to the geometric mean of three housekeeping genes (Gusb, Ywhz, and Gapdh). Three independent replicates were performed.

### 2.7 Supernatant collection and EV isolation

For supernatant collection, spheroids were cultured for 5 days, after which supernatant was collected from each well, centrifuged at 800 rpm for 5 min and at 2,000 rpm for 10 min, and stored at −80 °C until use.

For EV collection, OS-MSC spheroids from five independent replicates were cultured for 5 days in unbuffered acidic RPMI medium supplemented with 10% FBS depleted of EV (FDE); FDE was obtained via ultracentrifugation at 110,000 g overnight at 4 °C (Beckman Coulter, Milan, Italy).

EV pellets were obtained from the collected supernatant and purified by differential centrifugation: 500 *g* for 10 min (twice), 2,000 g for 15 min (twice), and 10,000 g for 30 min (twice) at 4 °C to remove floating cells and cellular debris. The supernatant was then ultracentrifuged at 110,000 g for 1 h at 4 °C (Beckman Coulter). The EV pellet was washed (110,000 g for 1 h at 4 °C), resuspended in PBS, and stored at −80 °C until use. Supernatant and EV protein amount was determined by BCA Protein Assay (Pierce).

### 2.8 Angiogenesis array

Supernatants or EVs collected from OS-MSC spheroids in unbuffered acidic condition treated with FTY720 were analyzed by Human Angiogenesis Array Kit (R&D Systems) according to the manufacturer instructions. EVs pellet was directly lysed with 100 μL of the Lysis Buffer (1% Igepal CA-630, 20 mM Tris-HCl (pH 8.0), 137 mM NaCl, 10% Glycerol, 2 mM EDTA, 10 μg/mL Aprotinin, 10 μg/mL Leupeptin hemisulfate, and 10 μg/mLPepstatin A). Protocol was followed according to manufacturer’s instructions. Chemiluminescent membrane development was performed in the BioSpectrum® Imaging System (UVP, Cambridge, United Kingdom). Detected proteins were quantified by the mean spot pixel densities from the array membrane using ImageJ software. Array was performed with two independent replicates.

### 2.9 Capillary western blotting

To extract proteins, cell spheroids were harvested and lysed using RIPA buffer (50 mM Tris-HCl, 150 mM NaCL, 1% Triton X-100, 0.5% Sodium deoxylcholate, 2% SDS, 0.5 mM EGTA and 10 mM NaF). EV pellet was directly lysed with RIPA buffer. Protein concentrations were analysed using BCA Protein Assay Kit (Pierce). All Western blots were performed using Simple Western (Bio-Techne) and following the instructions provided by the manufacturer. Three independent replicates were performed.

The primary antibodies and concentrations utilized were as follows: anti human CD-9 (Cell Signaling, Danvers, Massachusetts, United States) 1:10; anti human Flotillin-1 1:250 (Novus Biologicals, Centennial, Colorado, United States); anti human CD81 (System Biosciences, Palo Alto, CA, United States) 1:100; and anti human hsp70 (System Biosciences, Palo Alto, CA, United States) 1:100. Signal was quantified using the Compass software for Simple Western assays (Bio-Techne, https://www.bio-techne.com/resources/instrument-software-download-center/compass-software-simple-western).

### 2.10 Statistical analysis

Statistical analysis was performed with the GraphPad Prism 7 software (SAS Institute Inc.). Due to the small number of observations, data were not considered as normally distributed and non-parametric tests were used. The two-tailed Mann–Whitney U test was used for unpaired comparison of two independent variables. Differences between control and treated conditions across supernatant and EV groups were assessed by two-way analysis of variance (ANOVA). Data were expressed as mean ± standard error of the mean (SEM). Only p values <0.05 were considered for statistical significance.

## 3 Results

### 3.1 Acidosis stimulates endothelial proliferation and tubulogenesis in a tumor spheroid context

To confirm that tumor-associated acidosis promotes angiogenesis in our model, we first assessed the direct effect of acidic conditions on HUVEC proliferation and tubulogenesis. Acidic conditions were generated using unbuffered culture conditions, which allows the formation of a physiological intratumoral pH range (Avnet et al., 2021; Gillies et al., 1994), and were compared with neutral medium (pH 7.4). Acidified medium increased both endothelial proliferation and tubulogenesis (Supplementary Figures S1, S2). Tubule formation was closely monitored and quantified between 12 and 15 h post-seeding by analyzing the node number and total tubule length. This time window was selected based on the reproducible kinetics of tubulogenesis observed across experiments, rather than on a single arbitrary time point (Supplementary Figure S1).

**FIGURE 1:**
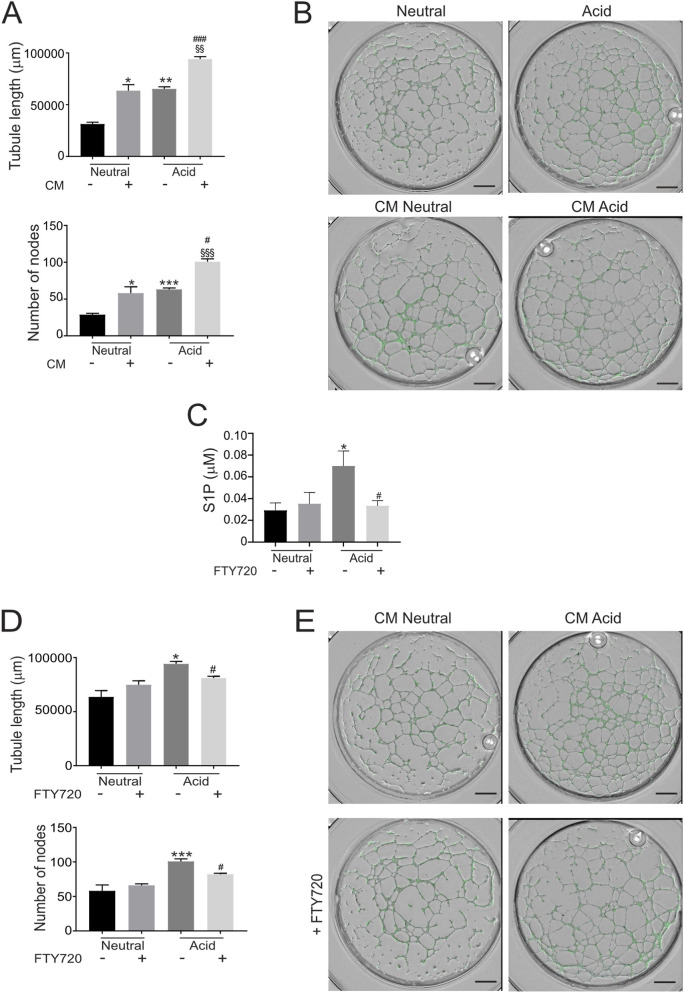
Acid-stimulated S1P, released by tumor cells, promotes tubulogenesis. **(A,B)** Quantifications and representative images of HUVEC-GFP cells treated with the CM of OS spheroids cultured in neutral or acidic conditions for 24 h (scale bar: 800 μm). The corresponding control (−) consisted of medium maintained at the same pH as that used for the spheroids. Mann–Whitney test (*p < 0.05 and **p < 0.01 versus neutral control; §§p < 0.01 vs. CM acid control; ###p < 0.005 vs. CM neutral control; n = 12). **(C)** S1P levels in CM of OS spheroids cultured for 96 h, at nuetral and acidic conditions, with or w/o FTY720 were determined by ELISA. Mann–Whitney test (*p < 0.05 vs. neutral control, #p < 0.05 vs. acid control). **(D,E)** Quantifications and representative images of HUVEC-GFP cells treated with the CM of OS spheroids, treated with FTY720, as indicated (scale bar: 800 μm). Mann–Whitney test (*p < 0.05 and ***p < 0.005 vs. neutral untreated control; #p < 0.05 vs. CM acid untreated control; n = 12). For all the experiments data are presented as mean ± SEM.

We next investigated whether acidosis also promotes angiogenesis indirectly through tumor-derived paracrine signals. To better model the TME, more faithfully than conventional 2D or homotypic tumor cell cultures, OS cells were co-cultured with AD-MSCs to generate 3D spheroids. This model incorporates tumor–stroma interactions and therefore better reflects the cooperative signaling processes that shape the angiogenic microenvironment. CM from acid-stimulated spheroids were then applied to GFP-expressing HUVEC to assess endothelial tubulogenesis. Notably, CM from spheroids exposed to acidic conditions elicited a substantially stronger pro-angiogenic response than extracellular acidosis alone, significantly increasing both total tubule length and branching node number compared with CM from spheroids maintained at neutral pH (Figures 1A,B; Supplementary Figure S3).

Collectively, these results indicate that extracellular acidosis promotes angiogenesis in this model predominantly through acid-induced changes in the spheroid secretome, rather than through a direct effect on endothelial cells.

### 3.2 S1P signaling contributes to acidosis-driven tubulogenesis

To determine whether S1P signaling contributes to the pro-tubulogenic activity of the tumor secretome under acidic conditions, we inhibited S1P signaling using FTY720 (Fingolimod), a functional antagonist of S1P receptors. Effective inhibition of S1P synthesis and secretion was first confirmed under our experimental conditions (Figure 1C). CM collected from FTY720-treated OS spheroids under acidic conditions exhibited a significantly reduced ability to promote endothelial tubule formation and branching, as demonstrated by a marked decrease in total tubule length and node number (Figures 1D,E). In contrast, direct treatment of endothelial cells with FTY720 in the absence of tumor CM did not significantly affect tubulogenesis, although a mild inhibitory trend was observed (Supplementary Figure S4). Importantly, endothelial cells directly exposed to FTY720 were likely subjected to higher effective drug concentrations than those present in CM, where the compound had already been metabolized by tumor cells.

These findings indicate that tumor-derived S1P signaling is required for the full pro-tubulogenic activity of the acid-conditioned tumor secretome, and that the observed endothelial responses are primarily mediated by tumor-derived factors rather than by direct effects of the inhibitor on endothelial cells.

### 3.3 S1P exerts step-specific effects on angiogenesis: inhibition of tubulogenesis and promotion of sprouting

**FIGURE 2:**
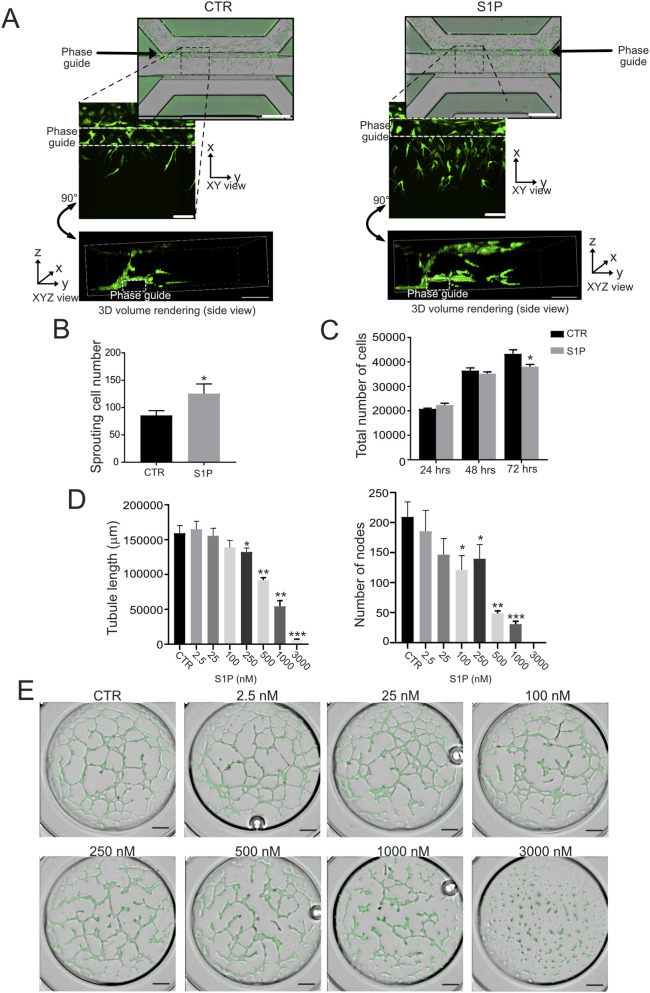
S1P stimulates endothelial cells sprouting. **(A)** Representative images of endothelial vessel formation after 24 h in a microfluidic platform, shown as whole-channel microscopy (Xpress Pico, upper panels) and high-magnification confocal microscopy (Nikon, lower panels) images. Three-lane Mimetas® microchambers were used, with HUVEC-GFP cells seeded in the top channel, Matrigel® loaded into the central channel, and the bottom channel reserved for the angiogenic cocktail (VEGF with or w/o 250 nM S1P). Dashed boxes highlight regions shown in the confocal close-ups. Confocal images display maximum-intensity projections and 3D side-rendered views. Scale bars: 50 μm. **(B)** Sprouting quantification (Mann–Whitney test; *p < 0.05 vs. control, n = 10). **(C)** HUVEC cell proliferation in neutral or acidic medium in monolayer for the indicated time points. Cells were treated with or w/o S1P added in the medium. The total number of cells was assessed by staining of cell nuclei by Hoechst. Data presented as mean ± SEM. Mann–Whitney U test (*p < 0.05 vs. control, n = 8). **(D,E)** Quantification and representative images of node number and total tubule length in HUVEC-GFP cells, treated with different S1P concentrations (nM), 12 h after seeding. (Mann–Whitney test; *p < 0.05 versus control, n = 9).

We next investigated the pro-angiogenic activity of S1P across distinct steps of angiogenesis under neutral conditions, to avoid confounding effects from other acid-induced pro-angiogenic mechanisms. We first evaluated endothelial sprouting in a physiologically relevant 3D context using a microfluidic device. In this model, GFP-expressing HUVEC were seeded in the upper channel and allowed to sprout into the extracellular matrix. Under these conditions, S1P significantly enhanced endothelial sprouting after 24 h, as demonstrated by live imaging, confocal microscopy, and quantitative analysis (Figures 2A,B). We then assessed subsequent stages of angiogenesis, including proliferation and tubulogenesis. In contrast to its pro-sprouting effect, S1P impaired endothelial cell proliferation (Figure 2C) and inhibited tubule network formation in a Matrigel-based assay in a dose-dependent manner (Spearman correlation, one-tailed, *p* = 0.0002 for tubule length and *p* = 0.0002 for number of nodes, Supplementary Figure S5). In particular, a significant reduction in node number was observed at 100 nM, while more pronounced inhibitory effects at 1 μM affected both node number and total tubule length and complete inhibition of tubulogenesis was observed at 3000 nM (Figures 2D,E). Time-course analysis up to 24 h revealed progressive regression of tubule structures at higher S1P concentrations (Supplementary Figure S6).

Together, these results reveal that S1P exerts context- and stage-dependent effects on angiogenesis, inhibiting endothelial network formation in a Matrigel-based morphogenesis assay while promoting invasive sprouting in a 3D microenvironment. This distinction underscores that S1P cannot be considered a uniformly pro-angiogenic factor, but rather a regulator of specific angiogenic steps.

### 3.4 Acidosis enhances the secretion of additional pro-angiogenic mediators beyond S1P

**FIGURE 3:**
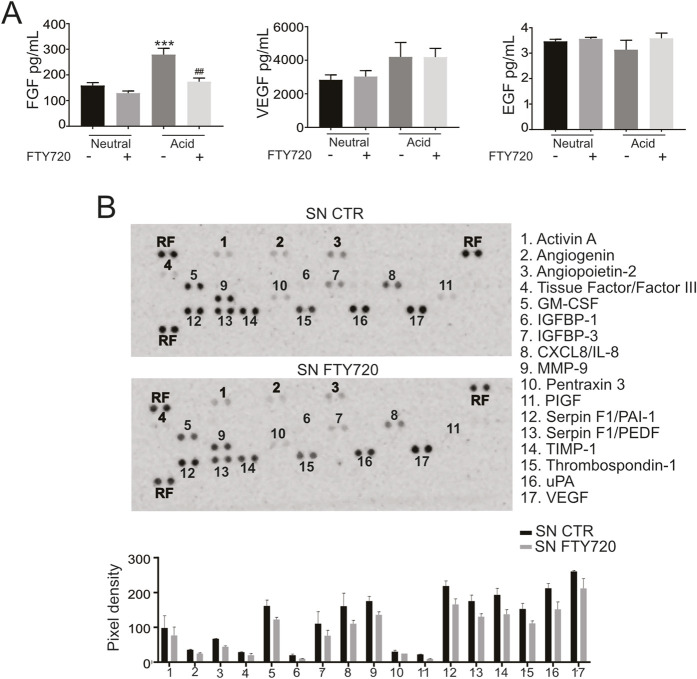
S1P promotes the secretion of pro-angiogenic soluble mediators. **(A)** FGF, VEGF and EGF levels in SN of OS spheroids cultured for 96 h treated with FTY720 were determined by ELISA. Mann–Whitney test (***p < 0.005 vs. neutral control, ##p < 0.01 vs. acid control). **(B)** Representative images and quantification of the positive spots associated with the angiogenesis-related proteins detected by the human angiogenesis array (RF: reference spot). Two-way Anova. For all the experiments data are presented as mean ± SEM, n = 2 independent experiments.

**FIGURE 4:**
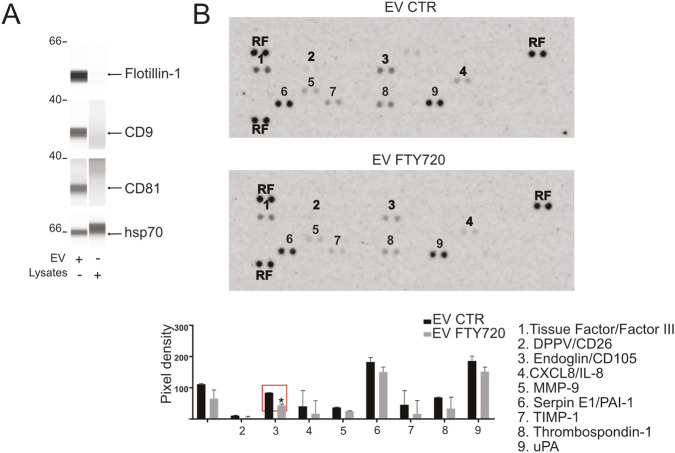
S1P promotes the secretion of EV-carried pro-angiogenic mediators **(A)** EV enrichment was assessed by capillary Western blot analysis for the expression of the specific EVs marker CD9, flotillin-1, CD81 and hsp70. **(B)** Representative images and quantification of the positive spots associated with the angiogenesis-related proteins detected by the human angiogenesis array (RF: reference spot). Data presented as mean ± SEM. Mann–Whitney test (*p < 0.05 vs. EV control, n = 2 independent experiments).

To further elucidate the mechanisms underlying the pro-angiogenic activity of acid-conditioned tumor spheroids, we analyzed the secretion of angiogenic mediators and their regulation by acid-induced S1P signaling. We focused on a panel of well-established angiogenic regulators, including bFGF, VEGF, and EGF. ELISA analysis revealed that bFGF levels were markedly increased in CM derived from acid-stimulated OS spheroids compared with neutral conditions, and this increase was strongly reduced upon FTY720 treatment, indicating an S1P-dependent mechanism (Figure 3A). In contrast, VEGF and EGF levels remained unchanged across conditions. To extend this analysis, we performed a membrane-based antibody array profiling a broader panel of angiogenesis-related proteins. Multiple mediators were detected, including activin A, angiogenin, angiopoietin-2, tissue factor (factor III), GM-CSF, IGFBP-1 and -3, CXCL8, MMP-9, pentraxin 3, PlGF, PAI-1, PEDF, TIMP-1, thrombospondin-1, uPA, and VEGF. Several of these factors exhibited a moderate but consistent reduction following FTY720 treatment (two-way ANOVA, p < 0.0001; Figure 3B), suggesting partial dependence on S1P signaling. Given that pro-angiogenic mediators can also be transferred via extracellular vesicles (EV), we next investigated whether S1P influences vesicle-mediated signaling. EVs isolated from CM were validated by enrichment of CD9, flotillin-1 and CD81 and absence of these markers in cell lysates, which confirmed successful EV isolation. In contrast, as expected, the heat-shock protein 70 (hsp70) was detected in all samples (Figure 4A). Antibody array profiling of EV cargo revealed a distinct proteomic profile compared to the soluble CM fraction. A general trend toward reduced protein levels was observed in EVs from FTY720-treated spheroids, particularly for pro-angiogenic factors such as tissue factor (Factor III), CD105 (Endoglin), Serpine E1 (PAI-1), and uPA. Among these, only CD105 was reduced to a significant extent in a clearly S1P-dependent manner (Figure 4B, red box).

Collectively, these findings demonstrate that tumor acidosis promotes a broad pro-angiogenic secretome, regulated in part by S1P but also involving additional S1P-independent mediators, highlighting the complexity of tumor-driven angiogenic signaling.

**FIGURE 5:**
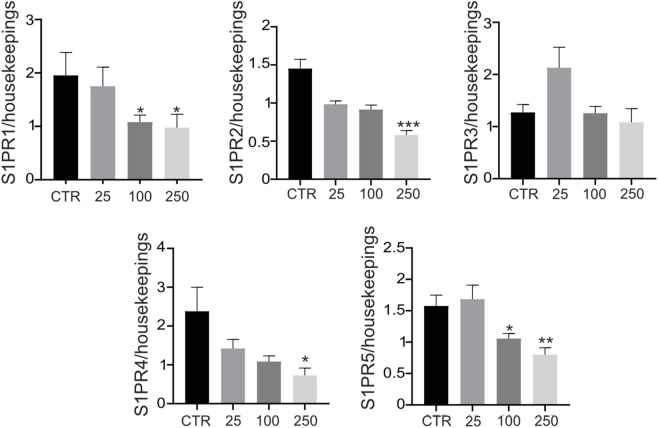
S1P modulates S1PRs expression. Real-time PCR analysis of the indicated genes normalized to three housekeeping genes (GAPDH, GUSB, and YWAHZ) in OS spheroids cultured for 72 h. Data presented as mean ± SEM. Mann–Whitney test (*p < 0.05 and **p < 0.01 vs. HUVEC vs. control; n = 9).

Finally, to further investigate S1P signaling regulation in tumor cells, we analyzed the expression of S1P receptors (S1PR_1–5_) in OS spheroids under basal conditions and following stimulation with increasing concentrations of S1P. Exogenous S1P treatment induced a general downregulation of S1P receptor expression, with limited receptor-specific exceptions (Figure 5), consistent with ligand-induced receptor desensitization mechanisms.

## 4 Discussion

**FIGURE 6:**
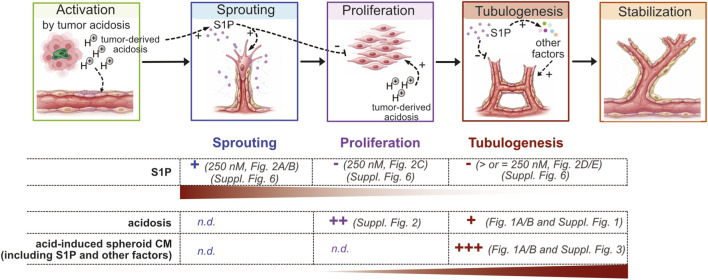
Schematic summary of the subsequent steps of angiogenesis and the main findings of this study. The table indicates the promoting or inhibitory effects of the different experimental conditions on angiogenic step. Tumor-associated extracellular acidosis reshapes the tumor secretome and enhances the release of S1P and other pro-angiogenic mediators, like bFGF and EV-associated factors, into the tumor microenvironment. S1P mainly promotes early angiogenic events, such as endothelial sprouting and migration, while impairing endothelial proliferation and tubulogenesis. In contrast, other components of the acid-induced secretome, support endothelial proliferation and tubular network formation. Together, these findings indicate stage-specific regulation of angiogenesis, in which S1P modulates early events, whereas additional mediators sustain later phases of vascular development (n.d., not determined).

Tumor angiogenesis is a highly coordinated, multistep process encompassing endothelial activation, sprouting, proliferation, tubulogenesis, and vessel stabilization (Liu et al., 2025) (Figure 6), each phase governed by distinct cellular programs and differentially regulated by the TME. Among the microenvironmental cues that orchestrate this process, extracellular acidosis, a hallmark of rapidly growing tumors, has emerged as a critical regulator of tumor–stromal interactions, including endothelial cell behavior (Corbet and Feron, 2017). Our findings place acidosis as an upstream organizer of a complex pro-angiogenic program in which S1P, consistent with its well-characterized pro-angiogenic role in cancer (Lee et al., 1999; Takuwa et al., 2010; Argraves et al., 2004), acts not as an isolated effector but as a central node within a broader signaling network (Figure 6).

Although HUVEC-based Matrigel®-based tubulogenesis assays remain robust and widely employed, they do not capture the tissue heterogeneity, such as that of the bone or lung microenvironment in OS (Boueya et al., 2025; Ramasamy, 2017; Shimizu and Kubota, 2025), nor the biochemical gradients and dynamic interactions of a perfused 3D setting. To partially address these limitations, we employed multicellular spheroids incorporating MSC, and perfused microfluidic platforms. MSC are recruited to acidic and inflamed tumor regions and contribute to tumor progression through ECM remodeling, neovascularization, and pre-metastatic niche formation, especially in the lung, the predominant metastatic site in OS (Cortini et al., 2023; Valenzuela Alvarez et al., 2024; Wang et al., 2024; Zheng et al., 2021). Microfluidic systems, in turn, enable controlled perfusion, spatial compartmentalization, and stable biochemical gradients, more closely recapitulating the *in vivo* vascular niche, including vascular barrier dynamics, and allowing assessment of endothelial sprouting under physiologically relevant flow conditions (Ozer et al., 2023; Soragni et al., 2024; Wevers et al., 2021).

Consistent with previous reports (Perut et al., 2019; Fang et al., 2008; Saghiri et al., 2020), extracellular acidosis directly increased endothelial proliferation, in contrasts to tumor cells, in which low extracellular pH is typically associated with growth inhibition (Avnet et al., 2025; Cortini et al., 2021). This divergence likely reflect cell type–specific adaptation to acidic stress, potentially involving Protein Kinase B (Akt) and Extracellular Signal-Regulated Kinase (ERK)-dependent signaling (Martinez et al., 2006). Importantly, however, the direct effect of acidosis on endothelial cells was modest compared with that elicited by CM from acid-exposed tumor spheroids, pointing to a key conceptual distinctions: acidosis does not merely act on endothelial cells directly, but reprograms the TME into a more potent angiogenic niche.

Within this acid-induced cascade, S1P emerges as a crucial mediator, consistent with our previous observations of enhanced S1P secretion from acid-stimulated OS spheroids and reduced circulating S1P levels in OS patients following standard therapy (Cortini et al., 2021). Our data reveal a stage-specific role for S1P: it preferentially enhanced early angiogenic events such as endothelial sprouting, as observed in the microfluidic assays, while failing to support, and at higher concentration (100 nM - 3 μM) imparing, tubule formation in Matrigel® assays in a dose-dependent way. However, this pattern is not consistent with previously described biphasic S1P responses (Kimura et al., 2000). The observed context dependence of S1P activity might also be shaped by receptor dynamics, microenvironmental conditions, and duration of ligand exposure. In this regard, we observed a general downregulation of S1P receptors following ligand stimulation, consistent with receptor desensitization through sustained ligand-induced internalization and functional uncoupling from downstream G-protein signaling (Juif et al., 2016; Xin et al., 2004). Notably, our experimental design was intentionally confined to the reported physiological range of S1P activity (Lee et al., 1999; Kimura et al., 2000; Bayless and Davis, 2003; Panetti et al., 2000; Williams et al., 2015), in contrast to studies employing supraphysiological concentrations (Kimura et al., 2000), in order to minimize non-specific effects that would complicate mechanistic interpretation.

Unexpectedly, however, FTY720 treatment, while exerting minimal direct effects on endothelial cells, consistently and significantly attenuated the tubulogenic capacity of tumor-derived acid-stimulated CM, supporting a role for S1P beyond direct endothelial cell sprouting stimulation, and suggesting S1P involvement in angiogenic signaling at multiple levels. Together, these findings suggest that S1P acts primarily as a priming signal for angiogenic initiation, while full vascular maturation depends on additional tumor-derived factors requiring coordinated cell–cell interaction and structural organization (Figure 6). Analysis of spheroid secretome confirmed that acidosis induces a broader pro-angiogenic program, that may involve both S1P-dependent and S1P-independent mediators. Among these, bFGF was significantly upregulated under acidic conditions, but it was also reduced upon S1P inhibition, indicating partial regulation by S1P signaling and further implicating this pathway in OS progression and angiogenesis (Huang et al., 2023; Li et al., 2019; Weekes et al., 2016). S1P-mediated indirect mechanisms on the secretion of pro-angiogenic factors, may also be independent to S1P receptors signalling since intracellular S1P has been shown to regulate histone deacetylases, TRAF2 (Camp et al., 2020; Mohammed and Harikumar, 2017), and intracellular calcium mobilization, mechanisms capable of sustaining tumor–endothelial communication independently of surface receptor engagment (Pulli et al., 2018). By contrast, VEGF and EGF secretion were not significantly altered by FTY720, suggesting that, unlike other tumor contexts (Krump-Konvalinkova et al., 2008; LaMontagne et al., 2006; Schmid et al., 2007), VEGF is unlikely to represent the principal effector of acidosis-induced angiogenesis in OS. At this point, it is important to acknowledge that the CM were derived from mixed OS- MSC spheroids, implying that angiogenic signatures reflect contributions from both tumor and stromal compartments. MSC are known to secrete pro-angiogenic factors (Aravindhan et al., 2021; Huang et al., 2013; Liang et al., 2021), mostly upregulated under acidic conditions (Avnet et al., 2017), and potentially establishing a reciprocal activation loop that reinforces angiogenic signaling within the TME. Acidosis may thus orchestrate a cooperative tumor–stroma program converging on S1P-dependent pathways. However, pathway attribution in the present study is based primarily on pharmacological modulation with FTY720. While the observed effects are consistent with involvement of S1P signaling, complementary genetic approaches targeting enzymes of the S1P metabolic or signaling pathways or specific S1P receptors will be important to further define receptor-specific and causal mechanisms.

Beyond soluble mediators, EVs, which are increasingly recognized as critical paracrine effectors of tumor-stromal communication and previously shown to transport pro-angiogenic miRNAs and proteins in OS under acidic conditions (Perut et al., 2019), also contributed to acidosis-driven angiogenic signaling. EV cargo profiling revealed a distinct set of angiogenesis-related proteins, several of which were reduced following FTY720 treatment. While these findings support a potential contribution of EVs to the pro-angiogenic phenotype, the present analyses are descriptive and do not establish EV cargo as a direct mechanistic driver of the endothelial responses observed. Among the angiogenesis-related proteins, Endoglin, a TGF-β co-receptor critical for vascular remodeling, was detected exclusively in the EV fraction and was significantly reduced after treatment. This is particular compelling given that Endoglin expression is upregulated under hypoxia, a major driver of tumor acidosis, and is supported by S1P/S1PR1 signalling in endothelial cells (Wang et al., 2022). Together, these observations suggest that S1P influences angiogenesis not only by inducing the secretion of soluble factors, but also by shaping EV-mediated delivery of TGF-β pathway components, thereby reinforcing vascular remodeling programs linked to the acidic TME (Rastogi et al., 2023).

Finally, it is worth noting that, as a clinically approved sphingosine analogue and functional S1P receptor antagonist, FTY720 is already clinically used in multiple sclerosis and is currently under clinical evaluation in breast carcinoma, glioblastoma, anaplastic astrocytoma, and lung cancer (NCT03941743, NCT02490930 and NCT06424067), underscoring the translational relevance of targeting this axis in aggressive malignancies such as OS.

As a limit of our study, although the integrated *in vitro* platforms employed here offer enhanced physiological relevance over conventional models, they cannot fully recapitulate the systemic complexity of *in vivo* tumor angiogenesis. Rather, they represent a step toward progressively more complex experimental frameworks, enabling mechanistic dissection under controlled conditions that would be difficult to resolve *in vivo*. Future studies could further expand this platform by incorporating additional components, such as endothelial tissue-specific subpopulations, immune cells, hypoxia, or *in vivo* validation models, to better reflect the full complexity of tumor angiogenesis.

Collectively, our findings support a model in which tumor acidosis promotes S1P release, which in turn amplify the tumor secretome, mostly via bFGF upregulation, and coordinates both soluble and EV-mediated pro-angiogenic signaling. Pharmacological disruption of this axis, particularly with clinically relevant agents like FTY720, may therefore represent a promising strategy to limit vascular support to aggressive OS.

## Data Availability

The raw data supporting the conclusions of this article will be made available by the authors, without undue reservation.
