# Supplementary material for: Tumor-derived sphingosine-1-phosphate shapes angiogenesis in the acidic microenvironment of osteosarcoma via paracrine and autocrine signaling
Source: Front Cell Dev Biol. 2026 May 21;14:1831997. doi: 10.3389/fcell.2026.1831997 (PMC13233457; doi:10.3389/fcell.2026.1831997)
Supplement: Supplementary file 1 [file Supplementaryfile1.pdf]

Supplementary Material

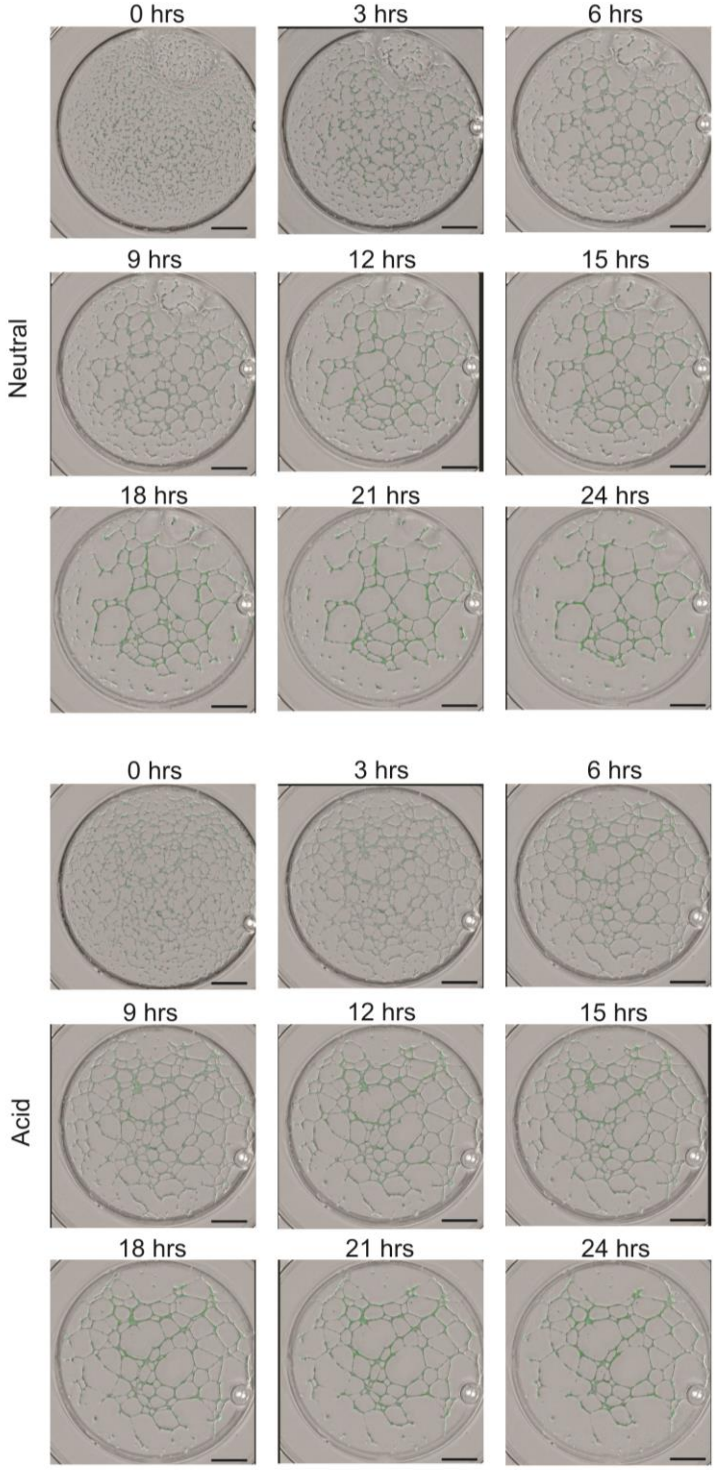

Supplementary Figure 1. Tubule formation over time in neutral and acidic conditions. Representative images of HUVEC-GFP cells cultured and assessed for 24 hours (scale bar: 800 μm).

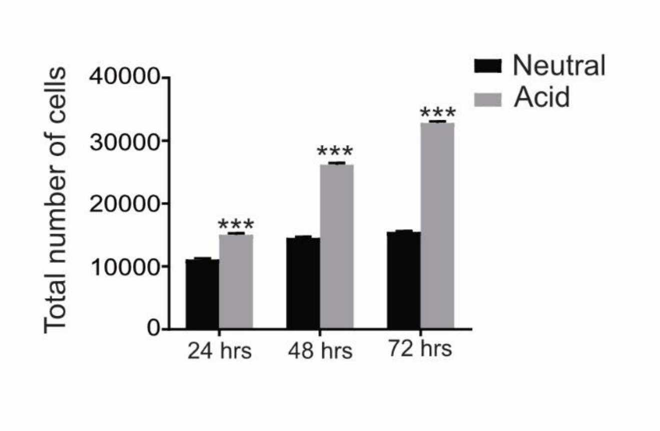

Supplementary Figure 2. HUVEC cell proliferation increases in acidosis. HUVEC endothelial cells were cultured in neutral or acidic medium in monolayer for the indicated time points. The total number of cells was assessed by staining of cell nuclei by Hoechst. Data presented as mean ± SEM. Mann–Whitney U test (***p < 0.005 vs control, n = 4).

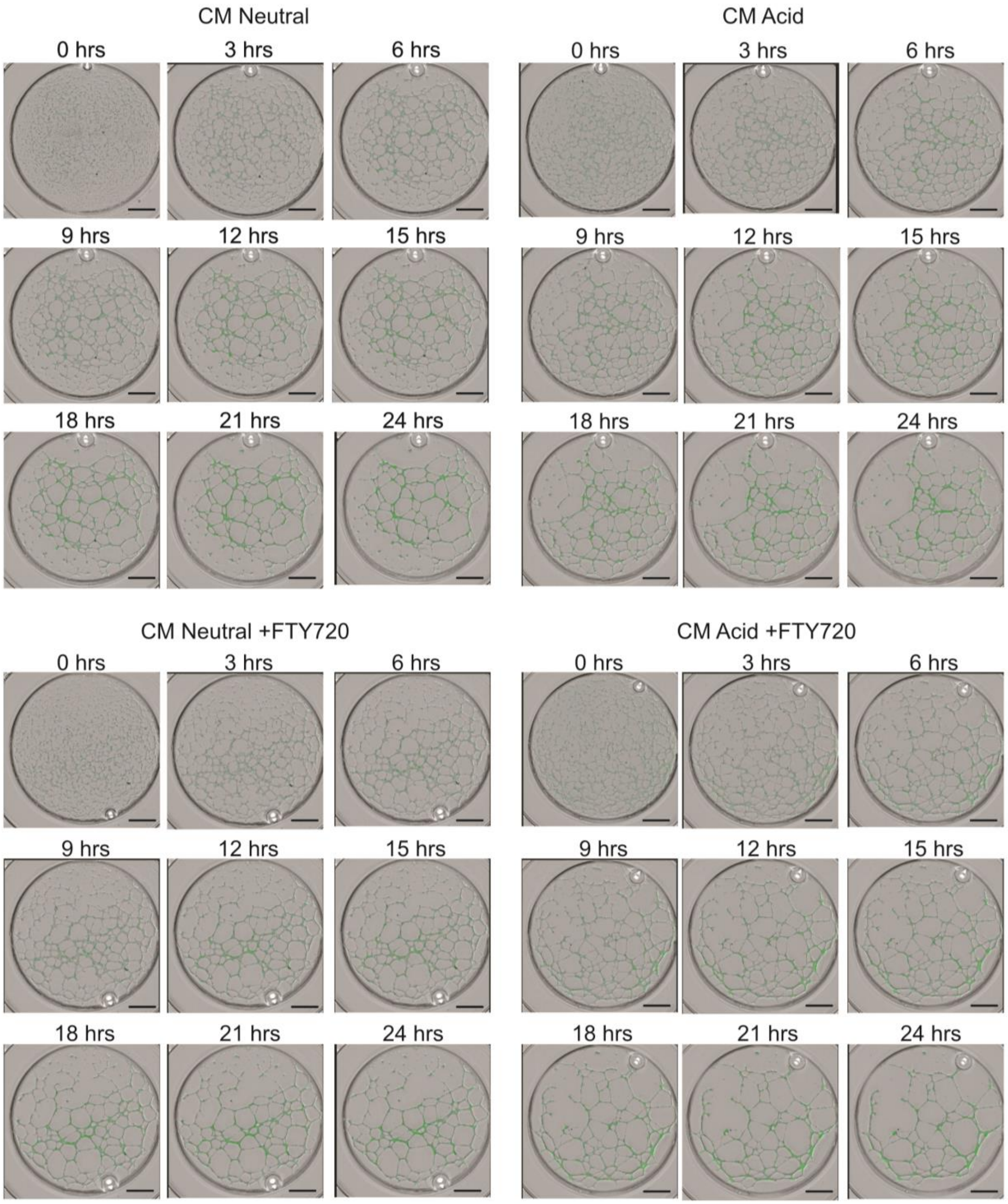

Supplementary Figure 3. Tubule formation over time after exposure to conditioned medium of OS spheroids, at neutral and acidic conditions. Representative images of treated HUVEC-GFP cells, cultured and assessed for 24 hours (scale bar: 800 μm).

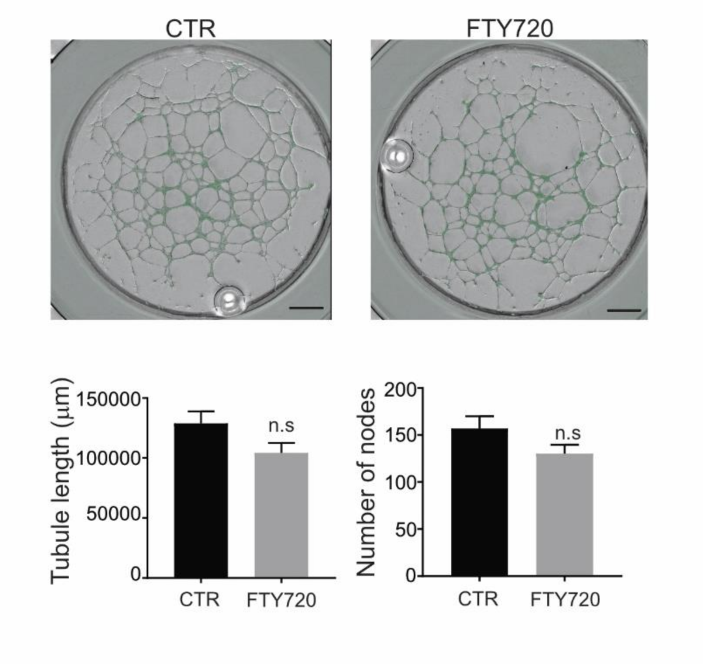

Supplementary Figure 4. Direct FTY720 exposure does not alter tubulogenesis. Quantification and representative images of HUVEC-GFP treated with FTY720 as indicated (scale bar: 800 μm). Data presented as mean ± SEM. Mann– Whitney U test (n = 12).

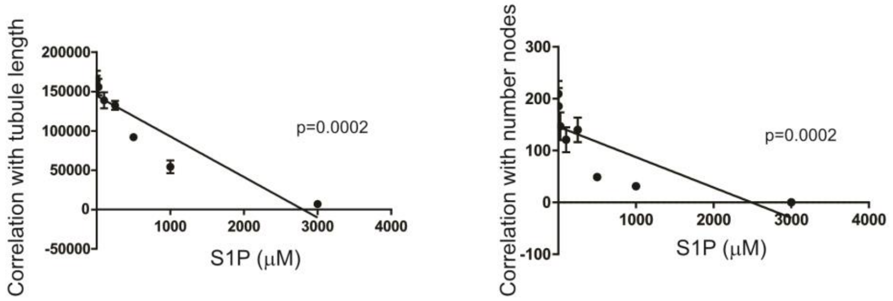

Supplementary Figure 5. Dose-response correlation between tubule length (left) and number of nodes (right) with increasing concentrations of S1P.

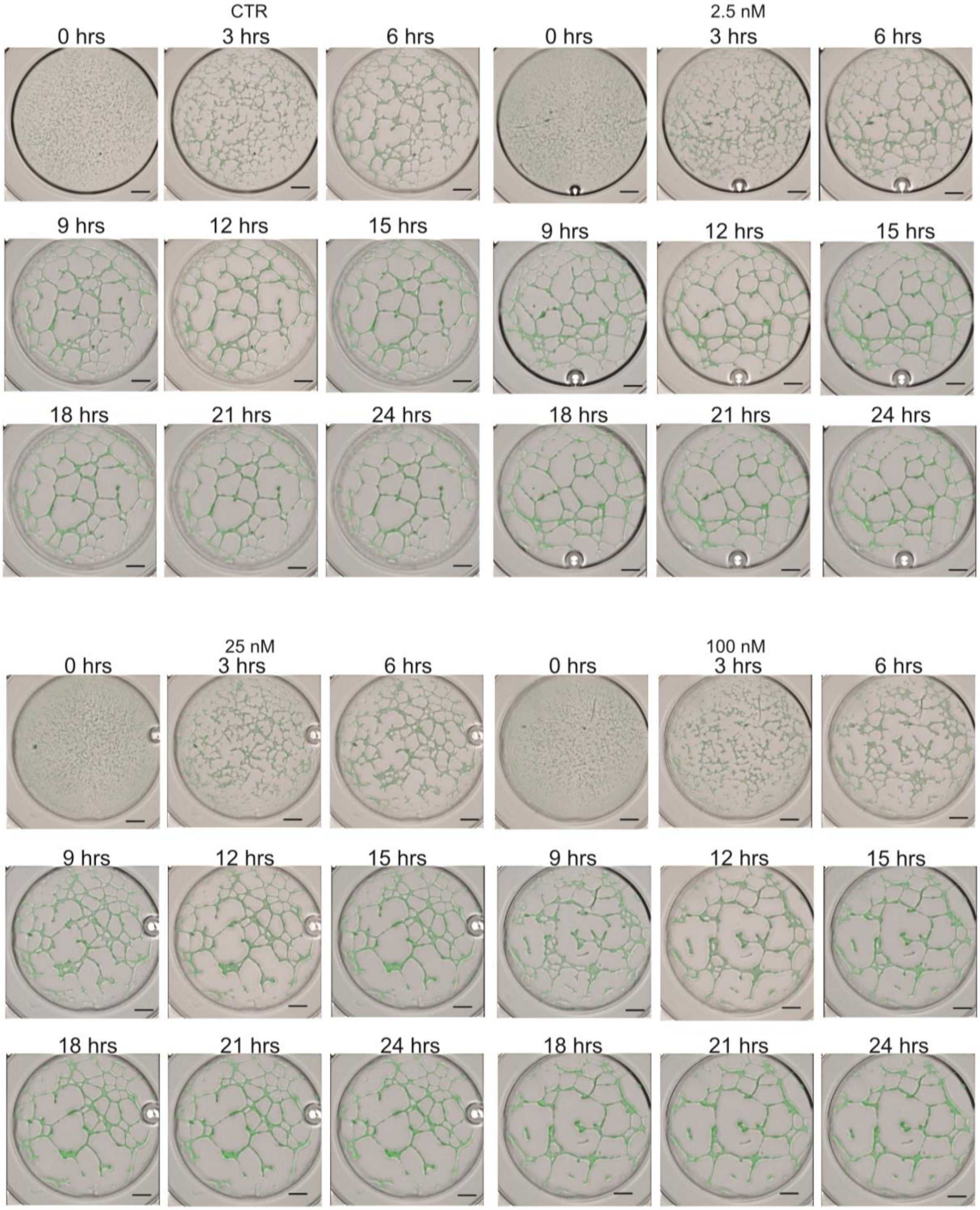
CTR
2.5 nM
0 hrs
3 hrs
6 hrs
9 hrs
12 hrs
15 hrs
18 hrs
21 hrs
24 hrs
25 nM
100 nM

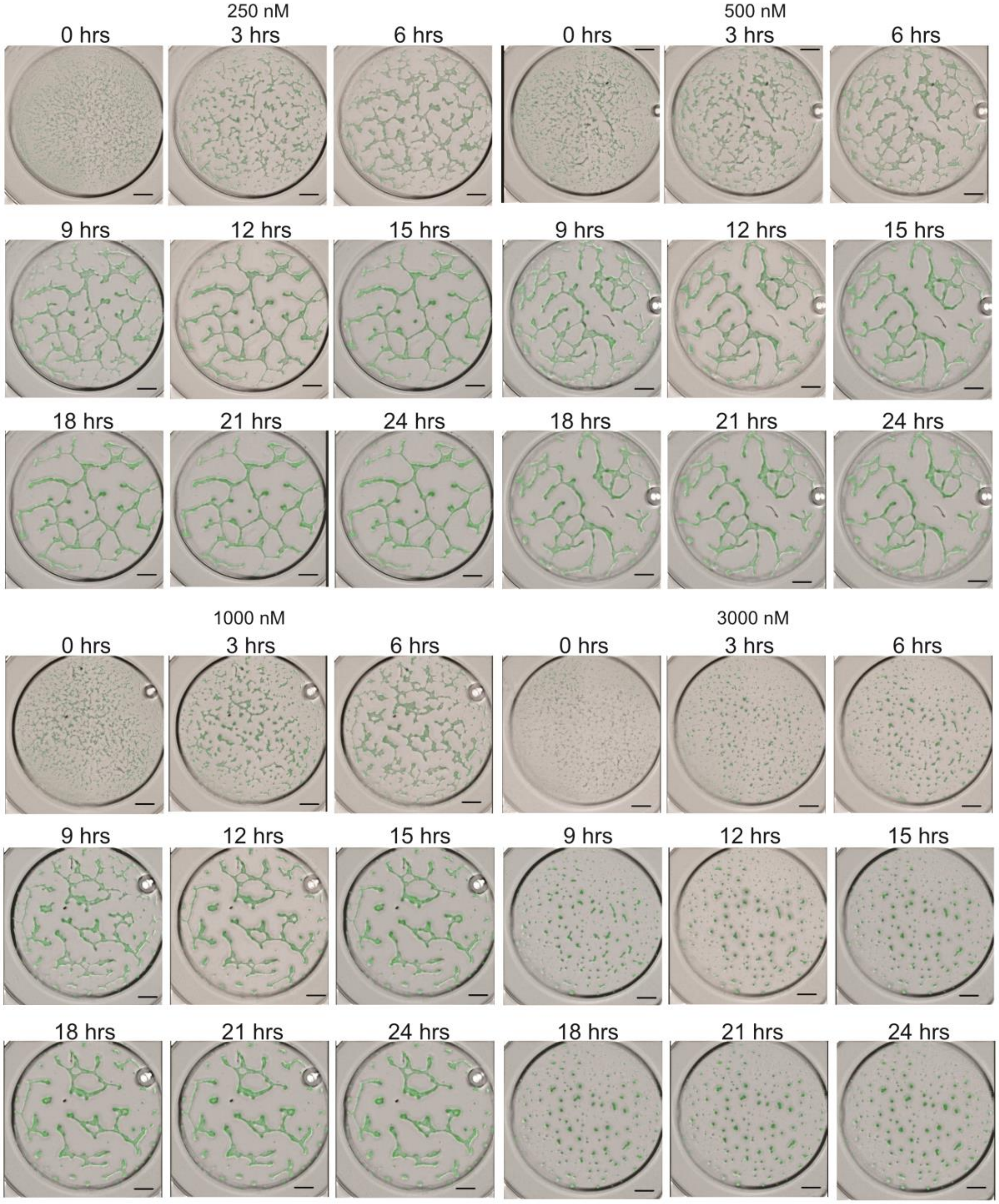

Supplementary Figure 6. Tubule formation over time. Representative images of HUVEC-GFP cells treated with the indicated doses of S1P, cultured and assessed for 24 hours (scale bar: 800 μm).
